# Network Pharmacology and In Vivo Evaluation of Modified Huanglian Wendan Decoction in Polycystic Ovary Syndrome With Insulin Resistance: Association With AGE–RAGE‐Related Signaling

**DOI:** 10.1002/cbdv.71737

**Published:** 2026-09-30

**Authors:** Xi Chen, Xing Yang, Xinni Wang, Dan Chou, Chenghong Zheng

**Affiliations:** ^1^ College of Traditional Chinese Medicine Hubei University of Chinese Medicine Wuhan China; ^2^ Department of Gynecology Wuhan Hospital of Traditional Chinese Medicine Wuhan China; ^3^ Wuhan Hospital of Traditional Chinese Medicine Wuhan China

**Keywords:** AGE–RAGE signaling, insulin resistance, Modified Huanglian Wendan Decoction, molecular docking, network pharmacology, polycystic ovary syndrome

## Abstract

Modified Huanglian Wendan Decoction (HLWDD) is used for polycystic ovary syndrome with insulin resistance (PCOS‐IR), but its biological basis remains incompletely defined. This study integrated network pharmacology, molecular docking, 100‐ns molecular dynamics (MD), batch‐specific chemical characterization, and in vivo evaluation in a letrozole/high‐fat diet rat model. HPLC fingerprinting showed high batch reproducibility, while UHPLC–HRMS/MS identified 997 candidate library assignments, including 118 higher‐confidence features. Network analysis identified 136 candidate compounds and 532 overlapping PCOS‐related targets, prioritizing AGE–RAGE signaling. Representative docked complexes remained stable during MD and showed negative MM/PBSA binding free energies. In vivo, HLWDD improved estrous‐cycle recovery and ovarian morphology, reduced fasting insulin, HOMA‐IR, reproductive hormones, and inflammatory markers, and modulated ovarian AGE, RAGE, STAT3, and AKT1 expression. High‐dose HLWDD also reduced p‐STAT3/STAT3 and increased p‐AKT/AKT1 relative to the Model group. These findings support an association between HLWDD treatment and AGE–RAGE/STAT3/AKT‐related signaling without establishing causal pathway mediation.

## Graphical abstract

An integrated network pharmacology, chemical profiling, molecular docking, molecular dynamics, and in vivo strategy shows that Modified Huanglian Wendan Decoction improves reproductive and metabolic abnormalities in a rat model of PCOS with insulin resistance. The findings implicate AGE–RAGE/STAT3/AKT1‐related signaling and provide a multi‐component framework for understanding the biological actions of this traditional formula.

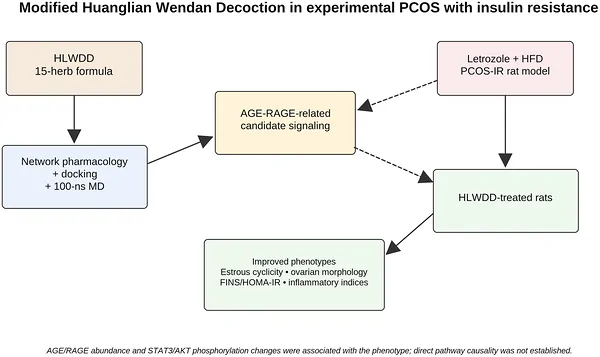

**Abbreviations**
AGE: advanced glycation end product
AKT1: AKT serine/threonine kinase 1
AMH: anti‐Müllerian hormone
FBG: fasting blood glucose
FINS: fasting insulin
GO: Gene Ontology
HLWDD: Modified Huanglian Wendan Decoction
HOMA‐IR: homeostatic model assessment of insulin resistance
HPLC: high‐performance liquid chromatography
IL‐6: interleukin‐6
IR: insulin resistance
KEGG: Kyoto Encyclopedia of Genes and Genomes
LH: luteinizing hormone
MD: molecular dynamics
MM/PBSA: molecular mechanics/Poisson–Boltzmann surface area
p‐AKT: phosphorylated AKT
PCOS: polycystic ovary syndrome
PPI: protein–protein interaction
p‐STAT3: phosphorylated STAT3
RAGE: receptor for advanced glycation end products
Rg: radius of gyration
RMSD: root–mean–square deviation
RMSF: root–mean–square fluctuation
SASA: solvent‐accessible surface area
STAT3: signal transducer and activator of transcription 3
T: testosterone
TCM: traditional Chinese medicine
TNF‐α: tumor necrosis factor‐α
UHPLC–HRMS/MS: ultrahigh‐performance liquid chromatography–high‐resolution tandem mass spectrometry

## 1 Introduction

Polycystic ovary syndrome (PCOS) is a common endocrine disorder of reproductive‐aged women, characterized by hyperandrogenism, ovulatory dysfunction, and polycystic ovarian morphology [1]. Reported prevalence varies according to diagnostic criteria [2]. Insulin resistance is frequent in PCOS and contributes to reproductive dysfunction and long‐term metabolic risk [3, 4]. Current management combines lifestyle intervention with pharmacological and ovulation‐induction therapies, but therapeutic response varies and the metabolic and reproductive components of the syndrome are often difficult to address simultaneously [4].

In traditional Chinese medicine (TCM), PCOS with insulin resistance is commonly discussed in relation to phlegm‐dampness or phlegm‐heat patterns [5, 6]. Modified Huanglian Wendan Decoction (HLWDD), derived from Huanglian Wendan Decoction, is used at Wuhan Hospital of Traditional Chinese Medicine and combines heat‐clearing and phlegm‐resolving herbs with other formula components. Experimental and network‐pharmacology studies of related prescriptions have suggested effects on metabolic, inflammatory, and insulin‐signaling processes [7, 8, 9, 10]. More broadly, TCM interventions have been discussed as modulators of endocrine and metabolic pathways relevant to PCOS [11], but the molecular basis of HLWDD in PCOS remains incompletely characterized.

Advanced glycation end products (AGEs) and their receptor RAGE have been implicated in PCOS‐related metabolic inflammation and ovarian dysfunction [12, 13, 14, 15]. AGE–RAGE signaling can interact with inflammatory pathways and with signaling processes involving STAT3 and PI3K/AKT [12, 13, 14, 15, 16]. These observations provide a biological rationale for examining AGE–RAGE‐related proteins in a PCOS‐IR model, while recognizing that this pathway represents only one component of a broader signaling network.

We therefore combined network pharmacology, molecular docking, and molecular dynamics (MD) with batch‐specific chemical characterization and in vivo evaluation in a letrozole/high‐fat diet rat model. The study aimed to characterize network‐predicted candidate compounds and targets, assess the dynamic stability of representative docked complexes, document the chemical profile of the administered HLWDD batch, evaluate reproductive, metabolic (including HOMA‐IR), inflammatory, and ovarian outcomes after HLWDD treatment, and determine whether ovarian AGE/RAGE abundance and STAT3/AKT1 abundance and phosphorylation readouts were associated with the observed phenotype. The experimental design was not intended to establish direct constituent‐target binding or causal pathway mediation.

## 2 Materials and Methods

### 2.1 Network Pharmacology, Molecular Docking, and Molecular Dynamics

#### 2.1.1 Screening of Database‐Derived Candidate Compounds and Targets

The chemical constituents of the 15 herbal materials in HLWDD (Table S1) were retrieved from the Traditional Chinese Medicine Systems Pharmacology Database and Analysis Platform (TCMSP) [17]. Candidate compounds were screened using oral bioavailability (OB) ≥ 30% and drug‐likeness (DL) ≥ 0.18. For compounds without target information in TCMSP, target information was supplemented from BATMAN‐TCM and literature records, guided by network‐pharmacology principles [18]. Protein names were harmonized to gene symbols and duplicate targets were removed. PCOS‐related targets were retrieved from GeneCards using the term “polycystic ovary syndrome” within the established diagnostic framework [19]; the 2000 highest‐relevance entries were used for intersection analysis. Overlapping HLWDD‐ and PCOS‐related targets were identified by Venn analysis.

#### 2.1.2 Network Construction and Pathway Analysis

Cytoscape 3.10.2 was used to construct the herb‐component‐target network [20]. A protein–protein interaction (PPI) network was generated using STRING with a confidence score >0.9 [21]. Hub genes were assessed using degree, betweenness, and closeness centrality. Gene Ontology (GO) and Kyoto Encyclopedia of Genes and Genomes (KEGG) enrichment analyses were conducted in R using clusterProfiler [22].

#### 2.1.3 Molecular Docking

Protein structures for AKT1 (PDB ID 7MYX), STAT3 (PDB ID 6NJS), and RAGE (PDB ID 6XQ1) were obtained from the RCSB Protein Data Bank, and three‐dimensional structures of the ten highest‐degree network‐ranked candidate compounds were obtained from PubChem. Protein and ligand preparation used AutoDockTools, and docking was performed with AutoDock Vina [23]. Docked complexes were inspected using UCSF Chimera [24]. All ten compounds were docked against all three targets. Docking scores were used only for comparative, hypothesis‐generating assessment and were not interpreted as proof of high‐affinity binding, stable interaction, target engagement, or in vivo constituent activity.

#### 2.1.4 Molecular Dynamics Simulation

Three representative docked complexes were selected for MD analysis based on the docking results and pathway focus: 4‐methylenemiltirone‐STAT3, 4‐methylenemiltirone‐AKT1, and Miltionone I‐RAGE. Simulations were performed using GROMACS 2025.2. Protein and ligand topologies were generated using CHARMM36 and GAFF parameters, respectively. Each complex was placed in a cubic periodic simulation box, solvated with the TIP3P water model, and neutralized with Na+ and Cl‐ ions. After energy minimization, NVT and NPT equilibration were each conducted for 2 ns using a 0.002‐ps time step. Production simulations were then run for 100 ns (50 000 000 steps) at 300 K and 1 bar. Trajectory analyses included root–mean–square deviation (RMSD), root–mean–square fluctuation (RMSF), radius of gyration (Rg), solvent‐accessible surface area (SASA), protein–ligand hydrogen‐bond counts, per‐residue MM/PBSA energy decomposition, and MM/PBSA binding free‐energy components.

### 2.2 In Vivo Experimental Evaluation

#### 2.2.1 Preparation, Batch‐Specific Chemical Characterization, and Dose Selection of HLWDD

HLWDD was prepared from 15 crude herbal materials dispensed by the Pharmacy of Wuhan Hospital of Traditional Chinese Medicine. The botanical names, medicinal parts, nominal amounts, and batch numbers of all components (total nominal crude‐herb amount, 151 g) are provided in Table S1. The herbs were soaked in 12 volumes of purified water for 30 min, heated to boiling, and decocted for 60 min. The residue was decocted a second time for 60 min with the same volume of purified water. The two filtrates were combined and concentrated under reduced pressure. The dry‐extract yield was 23.2% (w/w). The concentrated preparation was divided into sealed containers and stored at 4°C protected from light until use. Rat doses are reported as nominal crude‐herb equivalents. The medium dose (13.59 g/kg/d) was derived from the 151‐g/d adult prescription for a 70‐kg adult using the study protocol's conventional human‐to‐rat body‐surface‐area conversion coefficient of 6.3, consistent with body‐surface‐area scaling principles [25]. The low and high doses were 0.5‐ and 2.0‐fold the medium dose, respectively (6.80 and 27.18 g/kg/d). The high dose was therefore an exploratory preclinical dose rather than a clinical‐equivalent dose.

For batch‐specific ultrahigh‐performance liquid chromatography–high‐resolution tandem mass spectrometry (UHPLC–HRMS/MS) characterization, a 0.5–1.0‐mL aliquot of the same HLWDD preparation administered to the animals was mixed with two volumes of methanol/acetonitrile (1:1, v/v), vortexed for 60 s, sonicated at low temperature for 30 min, and centrifuged at 12 000 rpm for 10 min at 4°C. The supernatant was held at −20°C for 1 h and centrifuged again under the same conditions, vacuum‐dried, reconstituted in 100 µL of 50% acetonitrile, and centrifuged at 12 000 rpm for 10 min at 4°C before analysis. Chromatography was performed using a Vanquish UHPLC system coupled to a Q Exactive HFX Orbitrap mass spectrometer. Separation used a Waters HSS T3 column (100 × 2.1 mm, 1.8 µm) at 40°C, a flow rate of 0.3 mL/min, and a 2‐µL injection. Mobile phase A was 0.1% formic acid in water and mobile phase B was 0.1% formic acid in acetonitrile. The gradient was 0–1 min, 0% B; 1–12 min, 0%–95% B; 12–15 min, 95% B; 15–15.1 min, 95%–0% B; and 15.1–17 min, 0% B. Data were acquired separately in positive‐ and negative‐electrospray ionization modes using Full‐MS/ddMS2 over *m/z* 70–1050, with MS1 and MS2 resolutions of 70 000 and 17 500, respectively. Source settings were sheath gas, 40 arb; auxiliary gas, 10 arb; spray voltage, +3.0 kV/−2.8 kV; source temperature, 350°C; and ion‐transfer‐tube temperature, 320°C.

Raw UHPLC–HRMS/MS data were processed in Progenesis QI for baseline filtering, peak detection, retention‐time correction, and peak alignment. Candidate annotations were assigned using accurate mass and MS/MS spectral‐library matching; retention times were recorded for chromatographic characterization but were not interpreted as authentic‐reference‐standard confirmation. A higher‐confidence subset was defined by an MS/MS library‐match score ≥ 85 and an absolute mass error < 10 ppm. Relative peak‐area abundance was calculated as the integrated area of each feature divided by the summed integrated area of all features in the same ionization mode × 100. These values are mode‐specific semiquantitative ion abundances rather than absolute concentrations and are not comparable across ionization modes. The complete feature‐level dataset is provided in Data S1.

For orthogonal HPLC fingerprinting, an aliquot of the administered HLWDD batch was passed through a 0.22‐µm membrane and analyzed using an Agilent 1100 HPLC system with a variable‐wavelength detector. Separation was performed on a Thermo GOLD C18 column (250 × 4.6 mm, 5 µm) at 30°C with a 10‐µL injection volume, a flow rate of 1.0 mL/min, and detection at 230 nm. Mobile phase A was acetonitrile, and mobile phase B was 0.1% formic acid in water. The gradient was 0 min, 10% A/90% B; 40 min, 43% A/57% B; 45–50 min, 50% A/50% B; and 51 min, 10% A/90% B. Three replicate chromatograms from the same administered batch were compared with a reference chromatogram generated by the laboratory fingerprint‐matching workflow. The HPLC fingerprint and the positive‐ and negative‐ion total‐ion chromatograms are presented in Figures S1 and S2, respectively.

#### 2.2.2 Animals and Modeling

Fifty‐four specific pathogen‐free female Sprague‐Dawley rats (200 ± 20 g) were housed under standard conditions (22°C–27°C, 12‐h light/dark cycle). After acclimatization, rats were assigned to a Control group (*n* = 8) or a model‐induction cohort. PCOS‐IR was induced by daily gavage of letrozole (1 mg/kg in 1% CMC‐Na) combined with a high‐fat diet for 21 days [26, 27]. Modeling success was assessed before treatment allocation by persistent disruption of estrous cyclicity, defined as persistent diestrus with predominant leukocytes in vaginal smears. Of 46 rats undergoing model induction, 40 met this criterion and six were excluded before allocation to treatment groups. The model was selected to combine a reproductive PCOS‐like phenotype with a stronger metabolic component; no rodent model is assumed to reproduce the full heterogeneity of human PCOS.

#### 2.2.3 Grouping and Treatment

The 40 successfully modeled rats were randomly assigned to five groups (*n* = 8 each): Model (vehicle), Metformin (0.30 g/kg/d), HLWDD‐L (6.80 g/kg/d), HLWDD‐M (13.59 g/kg/d), and HLWDD‐H (27.18 g/kg/d). The Control group received vehicle. All treatments were administered by intragastric gavage once daily for 21 days.

#### 2.2.4 Estrous Cycle and Sample Collection

Vaginal smears were examined daily at 08:00 during the final 10 treatment days. A regular estrous cycle was defined as a 4–5‐day sequence progressing through proestrus, estrus, metestrus, and diestrus without persistent diestrus arrest. At the end of the experiment, rats were fasted for 12 h and anesthetized; blood was collected from the abdominal aorta and serum was separated by centrifugation (3500 rpm, 15 min, 4°C). Ovaries were collected for storage at −80°C or fixation in 4% paraformaldehyde.

#### 2.2.5 Biochemical Analysis

Serum luteinizing hormone (LH), testosterone (T), anti‐Müllerian hormone (AMH), fasting insulin (FINS), interleukin‐6 (IL‐6), and tumor necrosis factor‐α (TNF‐α) were measured using ELISA kits (Quanzhou Ruixin Biotechnology Co., Ltd., China) according to the manufacturer's instructions. Fasting blood glucose (FBG) was measured using a glucometer. Because paired individual‐animal FBG and FINS records were available, the homeostatic model assessment of insulin resistance (HOMA‐IR) was calculated for each rat as FBG (mmol/L) × FINS (mIU/L)/22.5.

#### 2.2.6 Histopathological Examination

Fixed ovaries were embedded in paraffin, sectioned at 4 µm, and stained with hematoxylin and eosin. Follicular development and ovarian morphology were assessed by light microscopy. Histological observations were qualitative; follicle counts and other morphometric endpoints were not prespecified for quantitative analysis.

#### 2.2.7 Western Blot Analysis

Ovarian tissue proteins were extracted using RIPA lysis buffer containing protease and phosphatase inhibitors. Protein concentration was determined by the BCA method. Equal protein amounts were separated by SDS‐PAGE and transferred to PVDF membranes. Total‐protein membranes were blocked with 5% nonfat milk, whereas phosphorylation blots were blocked with 1% BSA. Primary antibodies included AGE (A16422, ABclonal; 1:2000), RAGE (66833‐1‐Ig, Proteintech; 1:2000), STAT3 (60199‐1‐Ig, Proteintech; 1:1000), AKT1 (10176‐2‐AP, Proteintech; 1:2000), phospho‐STAT3 (Ser727; AP0715, ABclonal; 1:2000), phospho‐AKT (Ser473; 80455‐1‐RR, Proteintech; 1:1000), and GAPDH (60004‐1‐Ig, Proteintech; 1:20000). After incubation with HRP‐conjugated secondary antibodies, bands were visualized by enhanced chemiluminescence and quantified by densitometry. AGE, RAGE, STAT3, and AKT1 were normalized to GAPDH, whereas phosphorylation readouts were expressed as p‐STAT3/STAT3 and p‐AKT/AKT1 ratios. Densitometric comparisons were based on independent biological replicates and were analyzed using the statistical framework described below.

#### 2.2.8 Statistical Analysis

Continuous outcomes are presented as mean ± SD. Analyses were performed in SPSS 26.0. Variance homogeneity was assessed before group comparison. For outcomes with homogeneous variances, one‐way ANOVA followed by LSD post hoc testing was used; for outcomes with heterogeneous variances, Welch's ANOVA followed by Tamhane's T2 pairwise comparisons was used. The proportion of rats with a regular estrous cycle was compared with the Model group using two‐sided Fisher's exact tests. Western blot densitometry, including p‐STAT3/STAT3 and p‐AKT/AKT1 ratios, was analyzed using the same variance‐appropriate one‐way framework. HOMA‐IR was calculated from paired individual FBG and FINS values before group‐level analysis. All tests were two‐sided, and *p* < 0.05 was considered statistically significant. Body weight is presented at the prespecified study time points using group‐level summary statistics. Histopathological findings are interpreted qualitatively.

## 3 Results

### 3.1 Batch‐Specific Chemical Characterization of HLWDD

Batch‐specific UHPLC–HRMS/MS profiling generated 997 candidate library assignments, comprising 528 positive‐ion and 469 negative‐ion features; all had absolute mass errors < 10 ppm, and 118 additionally met the prespecified higher‐confidence criterion of an MS/MS library‐match score ≥ 85. Fifteen higher‐confidence, formula‐plausible candidates were selected for representative reporting, including palmatine chloride, icariin, coptisine chloride, narirutin, hesperidin, and nobiletin. Their mode‐specific relative peak‐area abundances are reported in Table S2, and the complete feature‐level dataset is provided in Data S1. Because electrospray response differs among compounds and ionization modes, the reported values are semiquantitative relative ion abundances rather than absolute chemical concentrations. The three HPLC replicate chromatograms showed similarities of 0.998, 1.000, and 0.999 to the laboratory reference chromatogram (Figure S1). Positive‐ and negative‐ion total‐ion chromatograms are shown in Figure S2. Constituent identities were not confirmed with authentic reference standards.

### 3.2 Network Pharmacology, Molecular Docking, and Molecular Dynamics

#### 3.2.1 Database‐Derived Candidate Compounds and Targets

A total of 136 database‐derived candidate compounds met the OB and DL criteria. TCMSP and BATMAN‐TCM‐based target collection yielded 1951 candidate compound‐related targets, and intersection with 2000 PCOS‐related GeneCards entries yielded 532 overlapping genes. Table 1 lists the ten highest‐degree candidate compounds and the ten highest‐degree targets in the herb‐component‐target network. The compound and target rankings are independent network summaries and do not imply a direct compound‐target relationship within each table row. These in silico candidates are distinct from, and should not be interpreted as, chemically confirmed constituents of the administered batch.

#### 3.2.2 PPI Network and Functional Enrichment

The PPI network comprised 476 nodes and 2158 edges. Gene Ontology enrichment highlighted processes related to gene‐expression regulation and responses to external stimuli. KEGG analysis identified AGE–RAGE signaling in diabetic complications among the enriched pathways (Figure 1). PI3K‐AKT, HIF‐1, and other inflammatory or metabolic pathways also appeared in the enrichment landscape and were not regarded as excluded. AGE–RAGE signaling was prioritized for targeted experimental follow‐up because it aligned with the metabolic‐inflammatory hypothesis and with the proteins measured in ovarian tissue.

**FIGURE 1:**
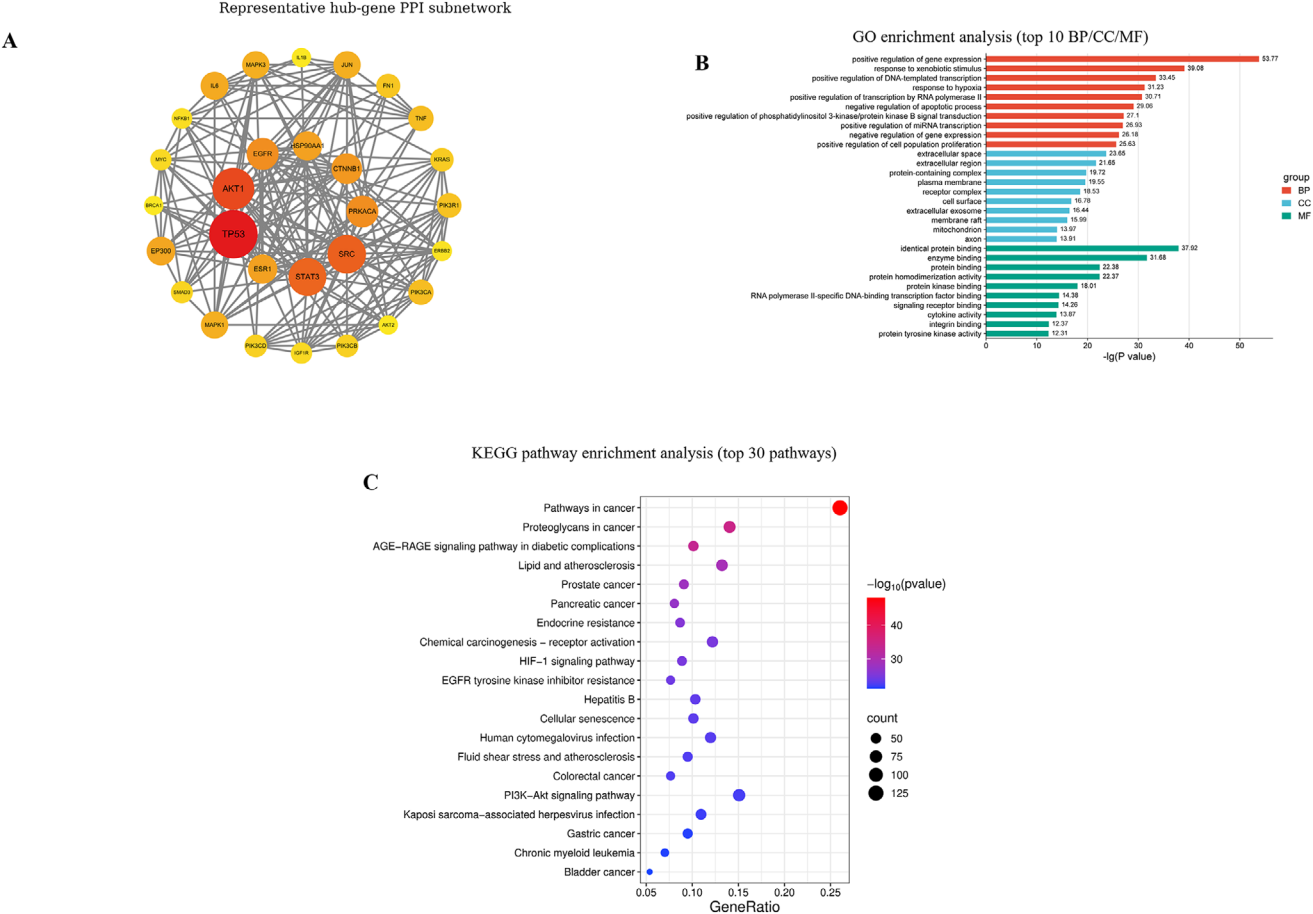
Network pharmacology analysis of HLWDD for PCOS. (A) Representative hub‐gene protein‐protein interaction subnetwork derived from the overlapping target set. (B) Gene Ontology enrichment analysis. (C) KEGG pathway enrichment analysis, including AGE–RAGE signaling in diabetic complications among the enriched pathways.

#### 3.2.3 Molecular Docking

The complete docking matrix for the ten network‐ranked candidate compounds against AKT1, STAT3, and RAGE is shown in Figure 2 and Table S3. Scores ranged from −1.0 to −7.3 kcal/mol. The most favorable values in the matrix included −7.0 kcal/mol for 4‐methylenemiltirone‐AKT1 and −7.3 kcal/mol for Miltionone I‐RAGE. Poricoic acid C, Gondoic acid, and Miltirone are shown together with the other candidates rather than being selectively omitted. These values represent computational predictions only; none of the individual compounds was administered or experimentally validated as an isolated active constituent in this study.

**FIGURE 2:**
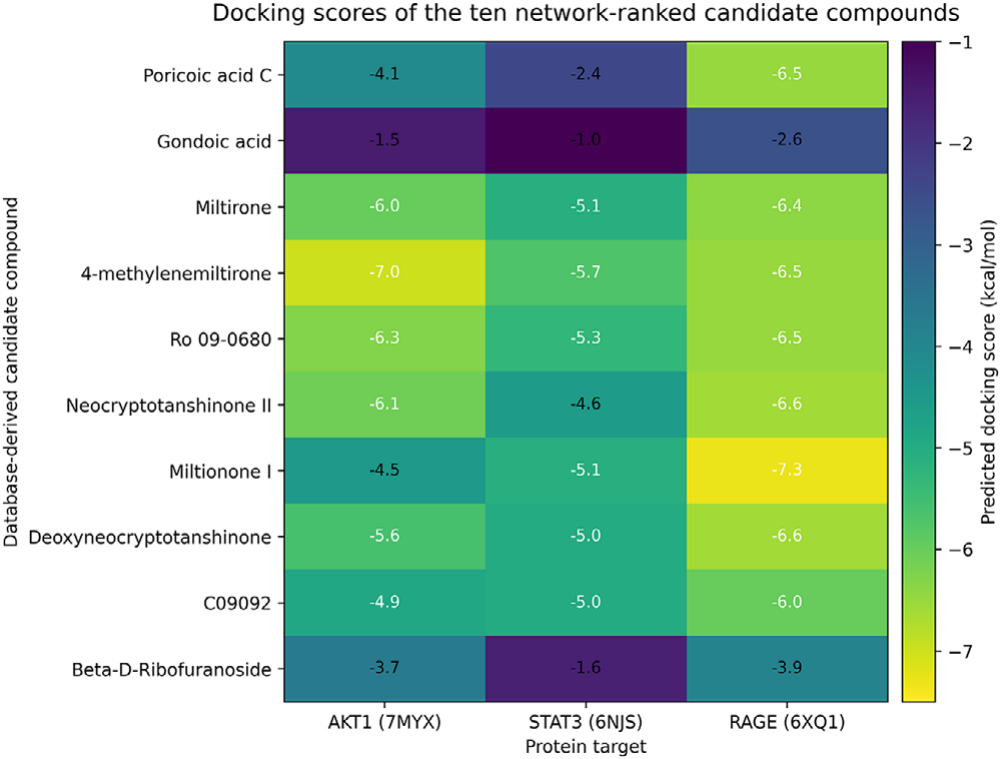
Molecular docking score matrix for the ten highest‐degree database‐derived candidate compounds and AKT1, STAT3, and RAGE. Values are predicted AutoDock Vina scores in kcal/mol; more negative values indicate more favorable predicted scores within this computational model. The matrix is presented for comparative hypothesis generation and does not establish high‐affinity binding, target engagement, or constituent‐level activity in vivo. Three representative complexes were subsequently evaluated by 100‐ns MD simulations (Figures S3–S6).

#### 3.2.4 Molecular Dynamics Simulation

The three representative protein–ligand complexes remained structurally stable during the 100‐ns production simulations, with protein RMSD values remaining below 2 Å throughout. For 4‐methylenemiltirone‐STAT3, RMSD was approximately 1.0–1.35 Å through most of 10–70 ns and reached a late maximum of about 1.55 Å; Rg remained at approximately 15.9–16.25 Å, SASA at 102–116 nm^2^, and hydrogen bonds were most commonly 1–3 with a transient maximum of 6. For 4‐methylenemiltirone‐AKT1, RMSD remained below 2 Å with a maximum of about 1.75 Å; Rg was approximately 15.9–16.35 Å, SASA 104–116 nm^2^, and hydrogen bonds were mainly 1–3 after the initial 20 ns with a maximum of 4. For Miltionone I‐RAGE, RMSD also remained below 2 Å; Rg was approximately 15.95–16.35 Å, SASA 102–116 nm^2^, and the trajectory generally retained a baseline hydrogen bond with transient increases to a maximum of 3. MM/PBSA analysis yielded negative total calculated binding free energies for all three complexes; van der Waals interactions were the dominant favorable component, whereas polar solvation opposed binding. Per‐residue energy decomposition identified Glu93, Pro74, and Tyr64 as the largest individual contributors in the STAT3, AKT1, and RAGE complexes, respectively. Detailed trajectories and energy analyses are shown in Figures S3–S6 and summarized in Table S4.

### 3.3 In Vivo Findings

#### 3.3.1 Body Weight and Estrous‐Cycle Recovery

Model rats showed greater body weight than Control rats after model induction. At Day 42, mean body weight was lower in each HLWDD‐treated group than in the Model group (Figure 3 and Table S5), although the pattern across the three HLWDD doses was not strictly monotonic. Control rats maintained regular 4–5‐day cycles, whereas Model rats showed persistent diestrus with predominant leukocytes in vaginal smears (Figure 4A). By the end of treatment, regular cycles were observed in 0/8 Model rats, 5/8 HLWDD‐L rats, 6/8 HLWDD‐M rats, 7/8 HLWDD‐H rats, 8/8 Control rats, and 8/8 Metformin rats. Two‐sided Fisher's exact comparisons versus the Model group are reported in Table S6. These findings describe recovery proportions and do not establish non‐inferiority or equivalence of HLWDD and Metformin.

**FIGURE 3:**
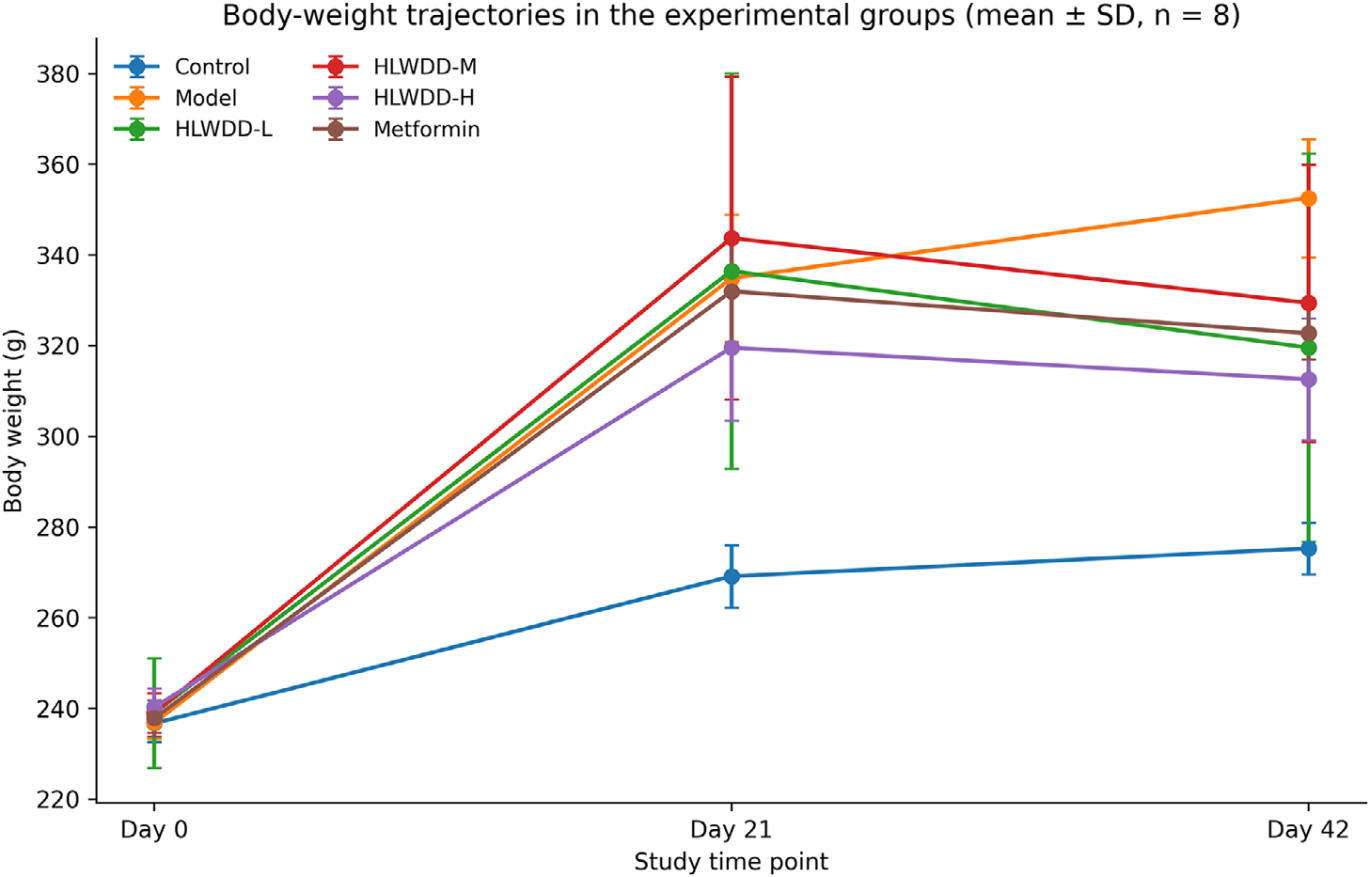
Body‐weight trajectories in the experimental groups. Values are group means ± SD (*n* = 8) at Day 0, Day 21 after model induction, and Day 42 after treatment. The figure is descriptive; complete group‐level data are provided in Table S5.

**FIGURE 4:**
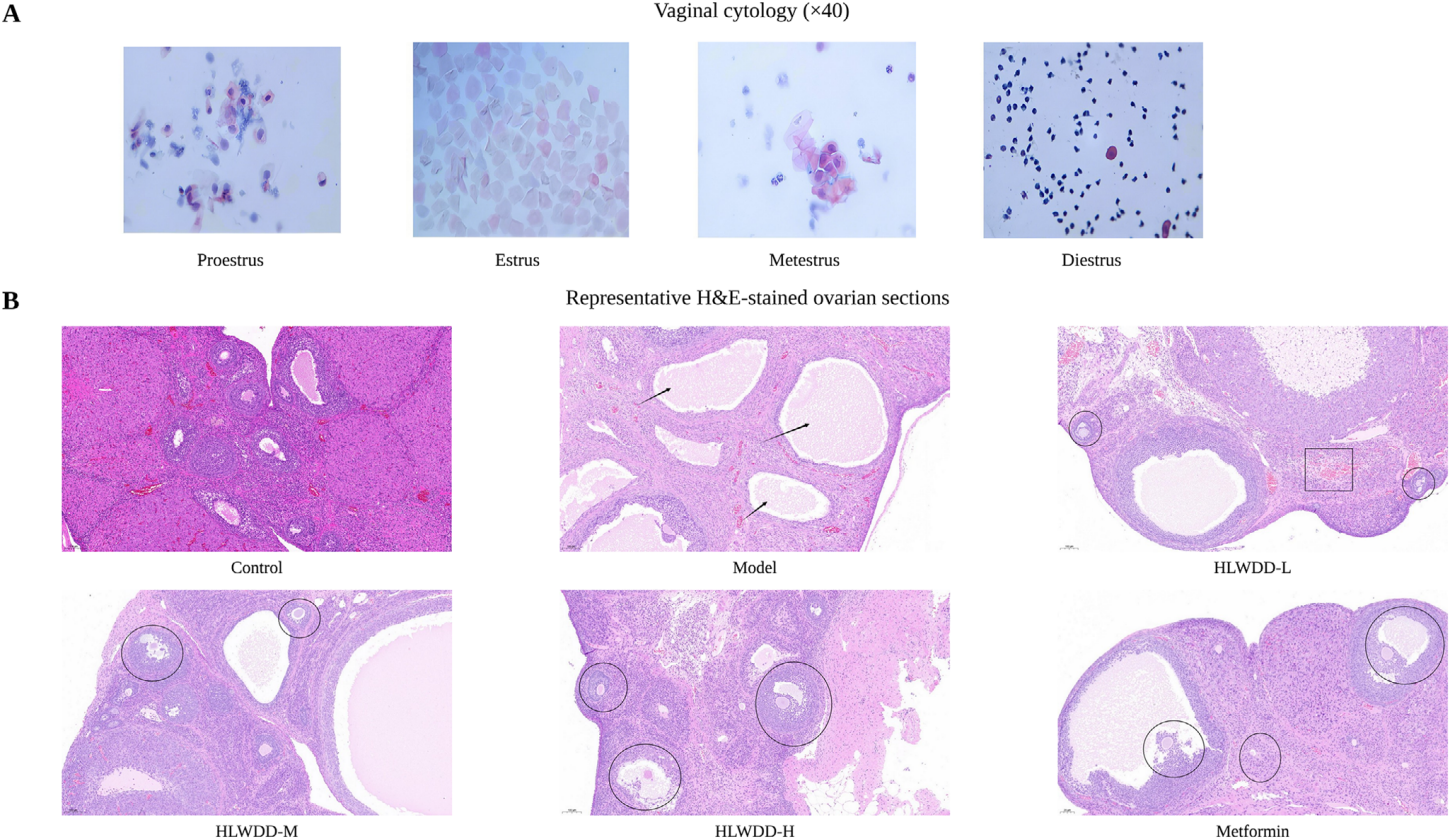
Histopathological evaluation of estrous‐cycle cytology and ovarian tissue. (A) Representative vaginal‐smear images illustrating proestrus, estrus, metestrus, and diestrus. (B) Representative H&E‐stained ovarian sections from Control, Model, HLWDD‐L, HLWDD‐M, HLWDD‐H, and Metformin groups. Histological findings were evaluated qualitatively.

#### 3.3.2 Qualitative Ovarian Histopathology

Control ovaries showed follicles at multiple developmental stages and corpora lutea. Model ovaries showed multiple cystic follicles, thickened theca layers, sparse granulosa cells, and few corpora lutea. HLWDD‐treated ovaries showed fewer cystic structures and more apparently healthy follicles and corpora lutea than Model ovaries (Figure 4B). Because morphometric endpoints were not quantified, these findings are interpreted qualitatively.

#### 3.3.3 Serum Hormonal, Metabolic, and Inflammatory Outcomes

Compared with Control rats, Model rats had higher T, LH, AMH, FINS, IL‐6, and TNF‐α values (all *p* < 0.01). Paired fasting measurements also showed a higher HOMA‐IR in the Model group (2.94 ± 0.65) than in the Control group (1.45 ± 0.08; Tamhane's T2 *p* = 0.0048). FBG alone did not differ significantly between Model and Control rats. Relative to the Model group, FINS was lower in all three HLWDD groups and in the Metformin group (all *p* < 0.01). HOMA‐IR was also lower after treatment: HLWDD‐L, 1.58 ± 0.11 (*p* = 0.0081); HLWDD‐M, 1.80 ± 0.44 (*p* = 0.0207); HLWDD‐H, 1.66 ± 0.40 (*p* = 0.0082); and Metformin, 1.54 ± 0.30 (*p* = 0.0043), each versus Model by Tamhane's T2. Pairwise FBG differences versus the Model group were not significant after variance‐robust multiple‐comparison adjustment. For the remaining hormonal and inflammatory outcomes, the low‐ and medium‐dose HLWDD groups showed significant reductions relative to Model, with larger differences generally observed in the high‐dose HLWDD and Metformin groups (Table 2). The serum findings therefore support improvement in insulin‐related, reproductive, and inflammatory endpoints without requiring a strictly monotonic dose‐response pattern.

#### 3.3.4 Ovarian AGE–RAGE/STAT3/AKT1 Protein Abundance and Phosphorylation Readouts

Western blot analysis showed higher ovarian AGE, RAGE, and STAT3 abundance and lower AKT1 abundance in the Model group than in the Control group (*p* < 0.01). High‐dose HLWDD was associated with lower AGE, RAGE, and STAT3 and higher AKT1 abundance relative to the Model group (*p* < 0.01). Phosphorylation analysis further showed that the p‐STAT3 (Ser727)/STAT3 ratio was higher and the p‐AKT (Ser473)/AKT1 ratio was lower in the Model group than in the Control group (both *p* < 0.01). High‐dose HLWDD significantly reduced p‐STAT3/STAT3 and increased p‐AKT/AKT1 relative to the Model group (both *p* < 0.01), and Metformin produced changes in the same direction (both *p* < 0.01 vs. Model). The low‐ and medium‐dose HLWDD groups showed intermediate directional changes (Figure 5; Tables 3 and S7). These total‐protein and phosphorylation findings strengthen the association of HLWDD treatment with AGE–RAGE/STAT3/AKT‐related signaling but do not establish causal pathway mediation.

**FIGURE 5:**
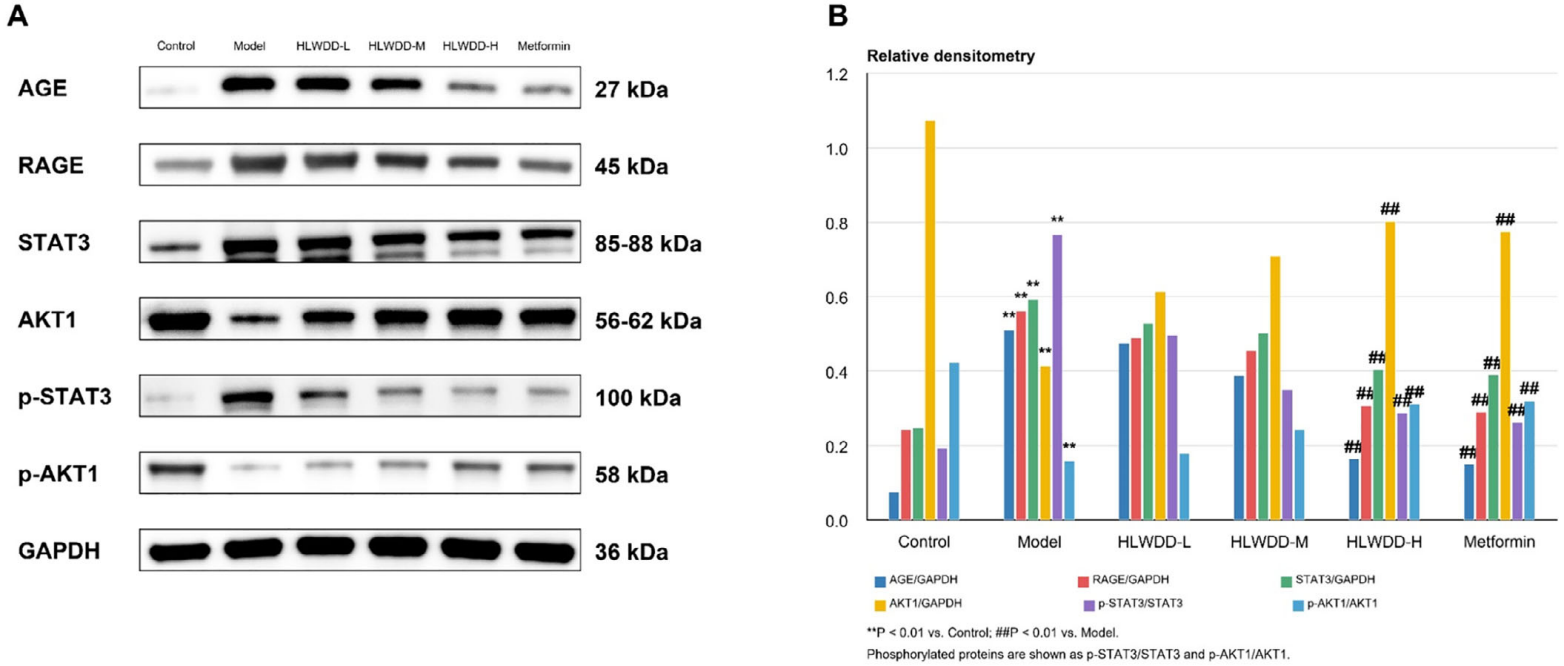
Ovarian AGE–RAGE/STAT3/AKT1‐related protein expression and phosphorylation. (A) Representative Western blot bands for AGE, RAGE, STAT3, AKT1, phospho‐STAT3 (Ser727), phospho‐AKT (Ser473), and GAPDH in the Control, Model, HLWDD‐L, HLWDD‐M, HLWDD‐H, and Metformin groups. (B) Relative densitometric analysis of AGE/GAPDH, RAGE/GAPDH, STAT3/GAPDH, AKT1/GAPDH, p‐STAT3/STAT3, and p‐AKT/AKT1. **p < 0.01 vs. Control; ##*p* < 0.01 vs. Model. Densitometric comparisons were based on independent biological replicates and analyzed as described in Methods.

## 4 Discussion

This study combined network pharmacology, molecular docking, 100‐ns MD simulations, batch‐specific chemical characterization, and an in vivo PCOS‐IR rat model to examine HLWDD‐associated reproductive, metabolic, inflammatory, and ovarian protein changes. Network‐pharmacology approaches have been applied to the study of multi‐component interventions in PCOS [28], while phlegm‐dampness‐related frameworks remain relevant to the TCM clinical context [29]. The computational analysis progressed from docking to dynamic evaluation of three representative complexes, all of which maintained protein RMSD below 2 Å and showed negative MM/PBSA total calculated binding free energies. The principal in vivo findings were improvement in estrous‐cycle recovery and ovarian morphology, marked reductions in fasting insulin and HOMA‐IR, changes in several hormonal and inflammatory indices, changes in total ovarian AGE, RAGE, STAT3, and AKT1 abundance, and significant phosphorylation‐level changes in STAT3 and AKT toward the Control pattern. These findings are interpreted as convergent computational and pathway‐associated evidence rather than proof of direct binding or a causal mechanism.

The network‐pharmacology candidates prioritized by topology and docking were derived from public databases and should not be regarded as chemically confirmed constituents of the administered batch. Independently, the administered preparation was analyzed directly by HPLC fingerprinting and UHPLC–HRMS/MS. The three HPLC replicate chromatograms showed high similarity to the laboratory reference fingerprint, and UHPLC–HRMS/MS provided batch‐specific candidate annotations and mode‐specific semiquantitative relative abundances for representative formula‐associated features. These two analyses address different questions: network pharmacology generates candidate compound‐target hypotheses, whereas direct chemical analysis characterizes the preparation used in vivo. Because library matching does not establish pharmacological activity and no authentic reference standards were used, the detected features are described as putative formula‐associated candidates rather than validated active ingredients. Targeted standard‐based quantification and direct testing of experimentally observed constituents remain priorities for future work.

The letrozole/high‐fat diet model was selected to combine a reproductive PCOS‐like phenotype with a stronger metabolic component. The observed hyperandrogenic, metabolic, inflammatory, and ovarian abnormalities are consistent with reports from related experimental PCOS models [27, 30]. The recovered paired fasting dataset enabled animal‐level HOMA‐IR calculation. HOMA‐IR was significantly higher in Model than Control rats and significantly lower in all three HLWDD groups and the Metformin group than in Model rats, whereas FBG alone did not show significant Model‐versus‐Control or treatment‐versus‐Model pairwise differences after Tamhane's T2 adjustment. This pattern indicates that the insulin‐related metabolic effect in the present dataset was captured more consistently by fasting insulin and the composite HOMA‐IR index than by fasting glucose alone. HLWDD treatment was also associated with improvement in reproductive and inflammatory phenotypes, consistent with prior studies of modified HLWDD or other metabolic interventions in experimental PCOS [31, 32, 33]. The serum results should be interpreted according to the individual post hoc comparisons rather than as a strictly monotonic dose‐response relationship, and the histological observations remain qualitative because follicle counts and other morphometric endpoints were not prespecified.

The MD results provide a dynamic extension of the docking analysis. Across the three representative complexes, RMSD, Rg, SASA, and hydrogen‐bond trajectories remained compatible with overall structural stability during 100 ns, while MM/PBSA calculations produced negative total binding free energies and identified van der Waals interactions as the main favorable energetic component. These results reduce uncertainty associated with single‐pose docking and support the plausibility of the selected complexes. Nevertheless, MD and MM/PBSA remain computational approaches and do not demonstrate direct target engagement in biological systems; orthogonal biophysical or biochemical binding assays would still be required for that purpose.

AGE–RAGE signaling was prioritized because it was highlighted by KEGG enrichment and has biological relevance to PCOS‐related metabolic inflammation. AGE/RAGE‐related dysfunction and PI3K/AKT/STAT3‐associated processes have been linked to ovarian and metabolic phenotypes in clinical and experimental studies [14, 15, 34, 35, 36, 37]. In the present study, Model ovaries showed higher AGE, RAGE, and STAT3 and lower AKT1 abundance, whereas high‐dose HLWDD showed changes in the opposite direction. Phosphorylation‐level Western blotting provided a more direct readout of signaling state: the Model group showed higher p‐STAT3 (Ser727)/STAT3 and lower p‐AKT (Ser473)/AKT1 than the Control group (both *p* < 0.01), while high‐dose HLWDD significantly reversed both abnormalities relative to the Model group (both *p* < 0.01). Metformin showed comparable directional changes. These results strengthen the evidence that HLWDD treatment is associated with modulation of STAT3 and AKT phosphorylation in ovarian tissue. However, pharmacological inhibition, genetic perturbation, rescue experiments, and direct binding assays were not performed; therefore, the findings do not establish causal pathway mediation. The integration of the computational and in vivo observations is summarized in Figure 6.

**FIGURE 6:**
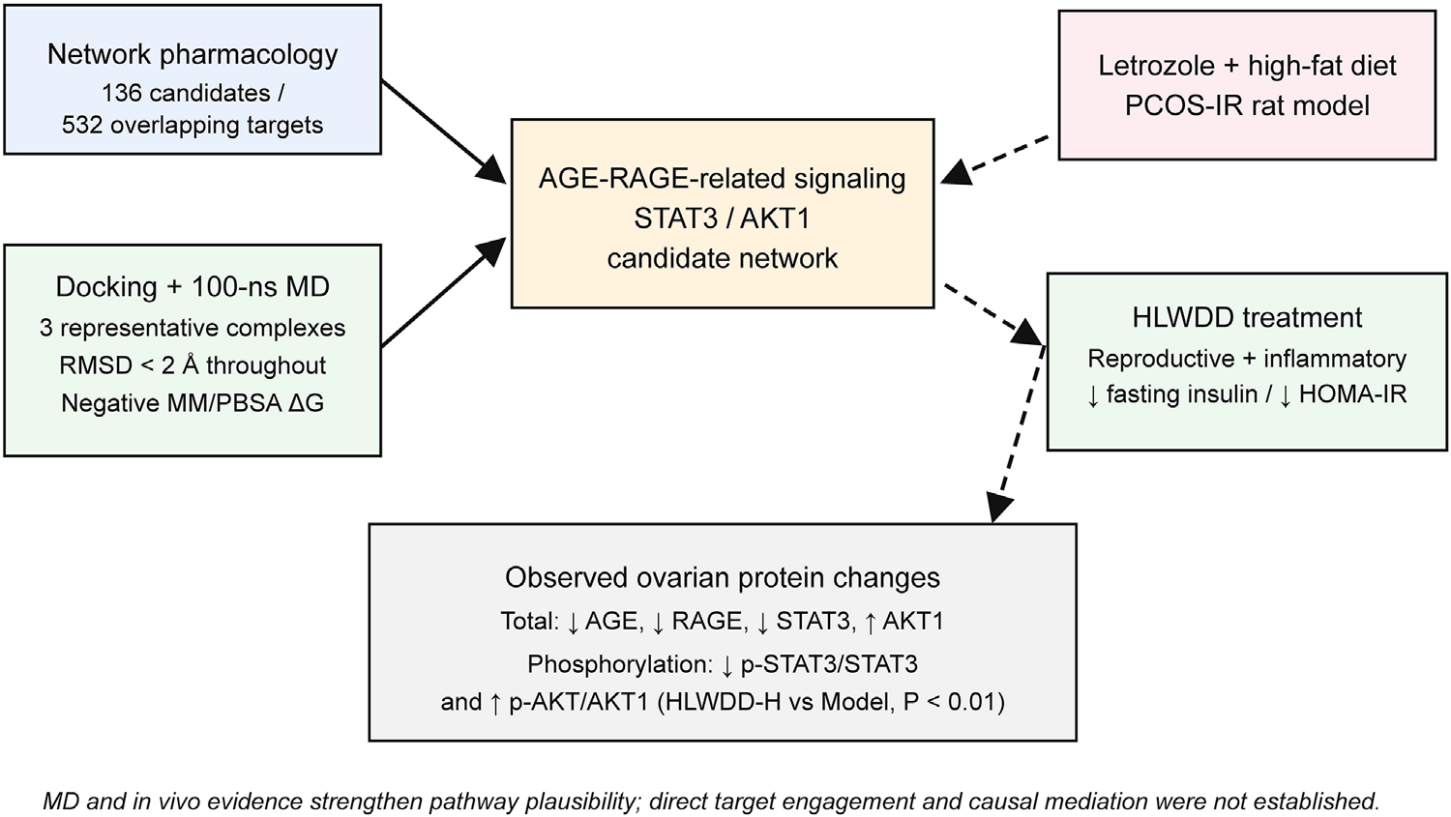
Working model integrating the computational and in vivo findings. Network pharmacology and docking prioritized AGE–RAGE/STAT3/AKT1‐related signaling as a candidate network; 100‐ns MD and MM/PBSA analyses supported the dynamic plausibility of three representative docked complexes. In vivo treatment was associated with reproductive, insulin‐related metabolic (including HOMA‐IR), inflammatory, total‐protein, and phosphorylation‐level changes. Dashed connectors denote association; direct target engagement and causal pathway mediation were not established.

Several limitations are important. First, the UHPLC–HRMS/MS assignments were library‐based and semiquantitative; they do not provide authentic‐reference‐standard confirmation, absolute quantification, or cross‐ionization‐mode comparability. Second, although 100‐ns MD and MM/PBSA analyses supported dynamic stability and favorable calculated binding energetics for three representative docked complexes, these remain computational observations; the database‐derived docking candidates were not experimentally integrated with the observed batch‐specific feature list, and orthogonal binding assays were not performed. Third, although phosphorylation‐level STAT3 and AKT measurements were added, pharmacological inhibition, genetic perturbation, rescue experiments, and direct binding assays were not performed, so causal pathway mediation cannot be established. Fourth, HOMA‐IR was calculated from paired fasting measurements but remains a surrogate index, and glucose‐tolerance or insulin‐tolerance testing was not performed. Fifth, ovarian histopathology was qualitative, and the study used a single animal model. Finally, the high dose of 27.18 g/kg/d was an exploratory crude‐herb‐equivalent dose and should not be interpreted as a corresponding human prescription. These limitations warrant targeted chemical, pharmacokinetic, mechanistic, dose‐ranging, tolerability, and safety studies before clinical translation.

## 5 Conclusion

HLWDD improved reproductive, insulin‐related metabolic, and inflammatory phenotypes in a letrozole/high‐fat diet rat model of PCOS‐IR, including significant reductions in fasting insulin and HOMA‐IR, and was associated with changes in ovarian AGE, RAGE, STAT3, and AKT1 protein abundance together with significant phosphorylation changes in STAT3 and AKT toward the Control pattern. Direct analysis of the administered batch provided a reproducible HPLC fingerprint and UHPLC–HRMS/MS candidate annotations, while 100‐ns MD and MM/PBSA analyses supported the dynamic plausibility of three representative docked complexes. The findings identify AGE–RAGE/STAT3/AKT‐related signaling as a candidate network for further investigation but do not establish direct target engagement or a causal multi‐target therapeutic mechanism.

## Author Contributions

**Xi Chen**: conceptualization, methodology, writing – original draft. **Xing Yang**: investigation and resources. **Xinni Wang**: formal analysis and visualization. **Dan Chou**: investigation and data curation. **Chenghong Zheng**: supervision and funding acquisition, writing – review and editing. All authors read and approved the final manuscript.

## Funding

This work was supported by the Traditional Chinese Medicine Innovation and Transformation Research Project of the China Center for Traditional Chinese Medicine Science and Technology Development (No. CXZH2025018); the Scientific Research Funding Project of the Wuhan Municipal Health Commission (Nos. WZ24Q23 and WZ24A04); the Wuhan Municipal Science and Technology Innovation Bureau (No. 2026020301040166); and the Hubei Provincial Department of Science and Technology (No. 2026AFC0865).

## Disclosure

A generative language model was used for language and structural editing. The authors reviewed and verified the scientific content, data, citations, interpretation, and final wording and take full responsibility for the manuscript. No generative image‐generation platform was used to create or modify the figures or graphical abstract.

## Ethics Statement and Consent to Participate

The study protocol was approved by the Ethics Committee of Hubei University of Chinese Medicine/Wuhan Hospital of Traditional Chinese Medicine (approval no. HUCMS69900002). All animal experiments were conducted in accordance with relevant guidelines and regulations for the care and use of laboratory animals.

## Conflicts of Interest

The authors declare no conflicts of interest.

## Supporting information




**Supporting File**: cbdv71737‐sup‐0001‐SuppMat.xlsx.

## Data Availability

The data reported in this study are presented in the article and Supporting Information, including the batch‐specific feature‐level UHPLC–HRMS/MS dataset. Additional study records can be requested from the corresponding author subject to institutional data‐retention and sharing requirements.

## References

[1] J. Bjekić‐Macut , T. Vukašin , Z. Velija‐Ašimi , et al., “Polycystic Ovary Syndrome: A Contemporary Clinical Approach,” Current Pharmaceutical Design 27, no. 36 (2021): 3812–3820. 10.2174/1381612827666210119104721 33463457

[2] Y. Chen , Q. Zhang , J. Ma , and Y. Yu , “Mapping Research Trends of Insulin Resistance in Polycystic Ovary Syndrome From 2017 to 2021: A Bibliometric Analysis,” Frontiers in Endocrinology 13 (2022): 963213. 10.3389/fendo.2022.963213 PMC9797656 36589816

[3] M. R. Somagutta , M. Jain , U. Uday , et al., “Novel Antidiabetic Medications in Polycystic Ovary Syndrome,” Discoveries 10, no. 1 (2022): e145. 10.15190/d.2022.4 PMC9745014 36518222

[4] H. J. Teede , C. T. Tay , J. J. E. Laven , et al., “Recommendations From the 2023 International Evidence‐Based Guideline for the Assessment and Management of Polycystic Ovary Syndrome,” European Journal of Endocrinology 189, no. 2 (2023): G43–G64. 10.1093/ejendo/lvad096 37580861

[5] Y. Li , H. Wu , Y. Guo , et al., “Cangfu Daotan Wan Alleviates Polycystic Ovary Syndrome With Phlegm‐Dampness Syndrome via Disruption of the PKP3/ERCC1/MAPK Axis,” Journal of Ovarian Research 16, no. 1 (2023): 134. 10.1186/s13048-023-01200-7 PMC10327183 37420272

[6] Q. Y. Zhao , Y. Sun , J. Zhou , et al., “Effectiveness of Herb‐Partitioned Moxibustion Combined With Electroacupuncture on Polycystic Ovary Syndrome in Patients With Symptom Pattern of Kidney Deficiency and Phlegm‐Dampness,” Journal of Traditional Chinese Medicine 41, no. 6 (2021): 985–993. 10.19852/j.cnki.jtcm.2021.06.017 34939397

[7] Z. Su , Y. Li , Z. Zhou , et al., “Huanglian Wendan Decoction Alleviates Metabolic Syndrome by Reprogramming Macrophage Polarization: Evidence From Multi‐Omics Integration,” Phytomedicine 146 (2025): 157119. 10.1016/j.phymed.2025.157119 40795588

[8] Q. Jin , J. Li , G. Y. Chen , et al., “Network and Experimental Pharmacology to Decode the Action of Wendan Decoction Against Generalized Anxiety Disorder,” Drug Design, Development and Therapy 16 (2022): 3297–3314. 10.2147/DDDT.S367871 PMC9526509 36193286

[9] Z. Gong , R. Li , S. Chen , et al., “Study on the Mechanism of Huanglian Jiedu Decoction in Treating Dyslipidemia Based on Network Pharmacology,” Journal of Healthcare Engineering 2022 (2022): 2457706. 10.1155/2022/2457706 PMC9433299 36061816

[10] W. Xu , M. Tang , J. Wang , and L. Wang , “Identification of the Active Constituents and Significant Pathways of Cangfu Daotan Decoction for the Treatment of PCOS Based on Network Pharmacology,” Evidence‐Based Complementary and Alternative Medicine 2020 (2020): 4086864. 10.1155/2020/4086864 PMC7057008 32148541

[11] H. Chen , C. Deng , Z. Meng , and S. Meng , “Effects of TCM on Polycystic Ovary Syndrome and its Cellular Endocrine Mechanism,” Frontiers in Endocrinology 14 (2023): 956772. 10.3389/fendo.2023.956772 PMC10228207 37260441

[12] M. Yurt and H. Gökmen‐Özel , “Are Dietary and Serum Advanced Glycation End Products (AGEs) Potential Contributors to Inflammation in Women With Polycystic Ovary Syndrome?,” Journal of Clinical Medicine 14, no. 16 (2025): 5803. 10.3390/jcm14165803 PMC12387888 40869631

[13] J. M. K. Azhary , M. Harada , C. Kunitomi , et al., “Androgens Increase Accumulation of Advanced Glycation End Products in Granulosa Cells by Activating ER Stress in PCOS,” Endocrinology 161, no. 2 (2020): bqaa015. 10.1210/endocr/bqaa015 32020188

[14] C. Tatone , G. Di Emidio , M. Placidi , et al., “AGEs‐Related Dysfunctions in PCOS: Evidence From Animal and Clinical Research,” Journal of Endocrinology 251, no. 2 (2021): R1–R9. 10.1530/JOE-21-0143 34448729

[15] M. Mouanness , H. Nava , C. Dagher , and Z. Merhi , “Contribution of Advanced Glycation End Products to PCOS Key Elements: A Narrative Review,” Nutrients 14, no. 17 (2022): 3578. 10.3390/nu14173578 PMC9460172 36079834

[16] L. M. Zhao , X. L. Su , Y. Wang , G. R. Li , and X. L. Deng , “KCa3.1 Channels Mediate the Increase of Cell Migration and Proliferation by Advanced Glycation Endproducts in Cultured Rat Vascular Smooth Muscle Cells,” Laboratory Investigation 93, no. 2 (2013): 159–167. 10.1038/labinvest.2012.163 23212096

[17] J. Ru , P. Li , J. Wang , et al., “TCMSP: A Database of Systems Pharmacology for Drug Discovery From Herbal Medicines,” Journal of Cheminformatics 6 (2014): 13. 10.1186/1758-2946-6-13 PMC4001360 24735618

[18] A. L. Hopkins , “Network Pharmacology: The Next Paradigm in Drug Discovery,” Nature Chemical Biology 4, no. 11 (2008): 682–690. 10.1038/nchembio.118 18936753

[19] R. E./A.‐S. P. C. W. Group , “Revised 2003 Consensus on Diagnostic Criteria and Long‐Term Health Risks Related to Polycystic Ovary Syndrome (PCOS),” Human Reproduction 19, no. (1) (2004):41–47. 10.1093/humrep/deh098 14688154

[20] P. Shannon , A. Markiel , O. Ozier , et al., “Cytoscape: A Software Environment for Integrated Models of Biomolecular Interaction Networks,” Genome Research 13, no. 11 (2003): 2498–2504. 10.1101/gr.1239303 PMC403769 14597658

[21] D. Szklarczyk , J. H. Morris , H. Cook , et al., “The STRING Database in 2017: Quality‐Controlled Protein‐Protein Association Networks, Made Broadly Accessible,” Nucleic Acids Research 45, no. D1 (2017): D362–D368. 10.1093/nar/gkw937 PMC5210637 27924014

[22] T. Wu , E. Hu , S. Xu , et al., “clusterProfiler 4.0: A Universal Enrichment Tool for Interpreting Omics Data,” Innovation 2, no. 3 (2021): 100141. 10.1016/j.xinn.2021.100141 PMC8454663 34557778

[23] O. Trott and A. J. Olson , “AutoDock Vina: Improving the Speed and Accuracy of Docking With a New Scoring Function, Efficient Optimization, and Multithreading,” Journal of Computational Chemistry 31, no. 2 (2010): 455–461. 10.1002/jcc.21334 PMC3041641 19499576

[24] E. F. Pettersen , T. D. Goddard , C. C. Huang , et al., “UCSF Chimera–A Visualization System for Exploratory Research and Analysis,” Journal of Computational Chemistry 25, no. 13 (2004): 1605–1612. 10.1002/jcc.20084 15264254

[25] US Food and Drug Administration , “Guidance for Industry: Estimating the Maximum Safe Starting Dose in Initial Clinical Trials for Therapeutics in Adult Healthy Volunteers,” Silver Spring (MD): US Food and Drug Administration, (2005).

[26] S. Qi , H. Yu , W. Hu , D. Xia , Q. Shi , and K. Wang , “Evaluation of Representativeness and Sustainability of PCOS Rat Models Induced by Letrozole With or Without High‐Fat Diet,” Scientific Reports 15, no. 1 (2025): 30754. 10.1038/s41598-025-15026-4 PMC12371043 40841528

[27] R. Hu , Y. Huang , Z. Liu , et al., “Characteristics of Polycystic Ovary Syndrome Rat Models Induced by Letrozole, Testosterone Propionate and High‐Fat Diets,” Reproductive Biomedicine Online 50, no. 1 (2025): 104296. 10.1016/j.rbmo.2024.104296 39626468

[28] X. Li , I. Ullah , C. Hou , Y. Liu , and K. Xiao , “Network Pharmacology and Molecular Docking Study on the Treatment of Polycystic Ovary Syndrome With Angelica Sinensis‐Radix Rehmanniae Drug Pair,” Medicine 102, no. 46 (2023): e36118. 10.1097/MD.0000000000036118 PMC10659600 37986355

[29] N. Zhang , C. Li , Y. Guo , and H. C. Wu , “Study on the Intervention Effect of Qi Gong Wan Prescription on Patients With Phlegm‐Dampness Syndrome of Polycystic Ovary Syndrome Based on Intestinal Flora,” Evidence‐Based Complementary and Alternative Medicine 2020 (2020): 6389034. 10.1155/2020/6389034 PMC7545460 33062017

[30] S. F. Mirseyyed , S. Zavareh , M. Nasiri , and H. Hashemi‐Moghaddam , “An Experimental Study on the Oxidative Status and Inflammatory Levels of a Rat Model of Polycystic Ovary Syndrome Induced by Letrozole and a New High‐Fat Diet,” International Journal of Fertility & Sterility 18, no. 1 (2024): 45–53. 10.22074/IJFS.2023.1972296.1391 PMC10692749 38041459

[31] Z. Wu , T. Yang , and H. Ma , “Molecular Mechanism of Modified Huanglian Wendan Decoction in the Treatment of Polycystic Ovary Syndrome,” Medicine 102, no. 15 (2023): e33212. 10.1097/MD.0000000000033212 PMC10101291 37058016

[32] J. Wang , Z. Huang , Z. Cao , et al., “Loureirin B Reduces Insulin Resistance and Chronic Inflammation in a Rat Model of Polycystic Ovary Syndrome by Upregulating GPR120 and Activating the LKB1/AMPK Signaling Pathway,” International Journal of Molecular Sciences 25, no. 20 (2024): 11146. 10.3390/ijms252011146 PMC11508921 39456928

[33] D. Rakic , V. Jakovljevic , V. Zivkovic , et al., “Multiple Benefits of Empagliflozin in PCOS: Evidence From a Preclinical Rat Model,” Pathophysiology 31, no. 4 (2024): 559–582. 10.3390/pathophysiology31040041 PMC11503319 39449523

[34] R. Zhang , Y. Liu , J. You , and B. Ge , “Tanshinone IIA Inhibits Ischemia‐Reperfusion‐Induced Inflammation, Ferroptosis and Apoptosis Through Activation of the PI3K/Akt/mTOR Pathway,” Human & Experimental Toxicology 42 (2023): 9603271231180864. 10.1177/09603271231180864 37314409

[35] Z. Tang , G. Yang , Z. Liao , et al., “Tanshinone IIA Reduces AQP4 Expression and Astrocyte Swelling After OGD/R by Inhibiting the HMGB1/RAGE/NF‐κB/IL‐6 Pro‐Inflammatory Axis,” Scientific Reports 12, no. 1 (2022): 14110. 10.1038/s41598-022-17491-7 PMC9388613 35982135

[36] C. Si , N. Wang , M. Wang , Y. Liu , Z. Niu , and Z. Ding , “TMT‐Based Proteomic and Bioinformatic Analyses of human Granulosa Cells From Obese and Normal‐Weight Female Subjects,” Reproductive Biology and Endocrinology 19, no. 1 (2021): 75. 10.1186/s12958-021-00760-x PMC8135161 34016141

[37] Q. Zhang , Z. Yang , X. Ou , M. Zhang , R. Ji , and G. Wu , “Hesperidin Inhibits Oxidative Stress and Apoptosis of Granulosa Cells in Polycystic Ovarian Syndrome Through the JAK2/STAT3 and PI3K/AKT Pathways,” Phytotherapy Research 39, no. 11 (2025): 5174–5197. 10.1002/ptr.70100 41014201

